# Effects of Mechanical Compression on Chondrogenesis of Human Synovium-Derived Mesenchymal Stem Cells in Agarose Hydrogel

**DOI:** 10.3389/fbioe.2021.697281

**Published:** 2021-07-19

**Authors:** Yuxiang Ge, Yixuan Li, Zixu Wang, Lan Li, Huajian Teng, Qing Jiang

**Affiliations:** ^1^State Key Laboratory of Pharmaceutical Biotechnology, Department of Sports Medicine and Adult Reconstructive Surgery, Nanjing Drum Tower Hospital, The Affiliated Hospital of Nanjing University Medical School, Nanjing, China; ^2^Laboratory for Bone and Joint Disease, Model Animal Research Center, Nanjing University, Nanjing, China; ^3^Jiangsu Engineering Research Center for 3D Bioprinting, Nanjing, China

**Keywords:** chondrocytes, mechanical compression, chondrogenesis, cartilage repair, SMSCs

## Abstract

Mechanical compression is a double-edged sword for cartilage remodeling, and the effect of mechanical compression on chondrogenic differentiation still remains elusive to date. Herein, we investigate the effect of mechanical dynamic compression on the chondrogenic differentiation of human synovium-derived mesenchymal stem cells (SMSCs). To this aim, SMSCs encapsulated in agarose hydrogels were cultured in chondrogenic-induced medium with or without dynamic compression. Dynamic compression was applied at either early time-point (day 1) or late time-point (day 21) during chondrogenic induction period. We found that dynamic compression initiated at early time-point downregulated the expression level of chondrocyte-specific markers as well as hypertrophy-specific markers compared with unloaded control. On the contrary, dynamic compression applied at late time-point not only enhanced the levels of cartilage matrix gene expression, but also suppressed the hypertrophic development of SMSCs compared with unloaded controls. Taken together, our findings suggest that dynamic mechanical compression loading not only promotes chondrogenic differentiation of SMSCs, but also plays a vital role in the maintenance of cartilage phenotype, and our findings also provide an experimental guide for stem cell-based cartilage repair and regeneration.

## Introduction

Cartilage damage caused by osteoarthritis (OA) or traumatic injury has become a major clinical problem, affecting a large number of people worldwide. Damaged articular cartilage generally leads to the gradual degradation of hyaline cartilage, which hardly repairs spontaneously due to the lack of vascularity and cellularity (Muir, 1995; Williams et al., 2003). As the articular tissue has a limited capacity to regenerate, current treatment is inefficient and not able to restore the function and mobility of damaged cartilage (Smith et al., 2005). Surgical treatments such as microfracture and autograft cartilage transplantation have shown great improvement but still result in fibrillation of cartilage, lacking in function and mobility of native cartilage. In this manner, there is great demand for the advanced techniques for the regeneration of cartilage (Bedi et al., 2010).

Cell-based tissue engineering has been widely studied and considered as an encouraging treatment of damaged cartilage. However, autologous chondrocyte implantation (ACI) has its disadvantage as isolated chondrocytes have a limited ability to proliferate (Wakitani et al., 2002). Mesenchymal stem cells (MSCs) are widely recognized as a multipotent cell source as they are easy to isolate and have a high capacity of self-renewing and chondrogenic differentiation (Tuan et al., 2003). Among these, synovium-derived mesenchymal stem cells (SMSCs) are considered as an ideal cell resource for cartilage tissue engineering due to their higher chondrogenic potential compared with other kinds of tissue-derived MSCs such as bone marrow-derived mesenchymal stem cells (BMSCs) (Sakaguchi et al., 2005; Pei et al., 2008).

Cartilage tissue engineering involves seeding MSCs into scaffolds such as different kinds of hydrogels, which are able to promote the production of extracellular matrix (ECM) components in three-dimensional (3D) culture (Bedi et al., 2010). There are various kinds of hydrogels for cartilage tissue engineering, including sodium alginate and agarose. Agarose has been specifically effective in cultivating engineered cartilage constructs with analogous mechanical and biochemical properties compared with native articular cartilage (O’Connell et al., 2017). Cell-based scaffolds are implanted after preculture *in vitro* for a short time or cultured for a long period to form a tissue, which has more proteoglycan and collagen contents. Despite the promising future of MSCs in cartilage regeneration, the ability of MSCs to regenerate hyaline-like cartilage is limited in that MSCs are more likely to differentiate into hypertrophic phenotypes followed by mineralization, and are eventually replaced with bone, which is known as endochondral ossification (Pelttari et al., 2006; Mueller and Tuan, 2008). Meanwhile, it has been demonstrated that the mechanical properties of cell-based scaffolds are much lower compared with those of native chondrocytes, implying that further optimization is needed (Mauck et al., 2006).

Many biochemical approaches have been applied to improve the chondrogenic potential of MSCs, such as members of transforming growth factor-β (TGF-β) family. TGF-β has been proved to act as a vital role in chondrogenesis, which is most commonly used in chondrogenic differentiation and induction (Toh et al., 2005). On the other hand, it has been demonstrated that biophysical cues such as compressive strain play a central role in chondrogenic differentiation (Mouw et al., 2007; Li et al., 2012; O’Conor et al., 2013; McKee et al., 2017). Chondrocytes in articular cartilage are surrounded by ECM, mainly composed of collagen 2 and aggrecan. During the daily activity, articular cartilage is exposed to great compressive strain, transmitting physical signals through the articular cartilage tissue into chemical cues, including changes in cell morphology and local osmolarity (Schiavi et al., 2018).

Accumulating evidence has suggested that mechanical cues significantly influence ECM synthesis of chondrocytes and play an important role in the differentiation of MSCs (Haugh et al., 2011; Fahy et al., 2018; Chen et al., 2021). Although great efforts have been made to optimize loading parameters to maximize the mechanical regulation on chondrogenic differentiation, especially in frequency and magnitude, rare studies have focused on how and when the mechanical stimulation should be applied (Zuscik et al., 2008; Kisiday et al., 2009; Li et al., 2010). In most studies, mechanical stimulation was applied at the initiation or relative early stage of chondrogenic differentiation period. Huang et al. (2010) reported that dynamic compression for 7 days from the initiation of chondrogenic differentiation displayed higher levels of chondrogenic markers. However, it was reported that preculture for 16 days increased the expression of chondrogenic markers compared with preculture for only 8 days during the chondrogenic differentiation (Mouw et al., 2007). Haugh et al. (2011) also reported that dynamic compression applied at 3 weeks was found to promote cartilage-specific ECM gene expression. These studies suggest that the mechanosensitivity of stem cells may vary during the process of chondrogenic differentiation (Mouw et al., 2007; Yang et al., 2009). Moreover, it has been shown that allowing ECM accumulation by delaying the application of mechanical compression enhanced mechanical properties of cartilage constructs or sulfated glycosaminoglycan (sGAG) synthesis (Demarteau et al., 2003; Lima et al., 2007). Moreover, Aisenbrey et al. reported that mechanical loading inhibited hypertrophy in chondrogenic differentiation of MSCs (Aisenbrey and Bryant, 2016). In this study, SMSCs were allowed to undergo chondrogenic differentiation in the presence of TGF-β3 for 4 weeks and dynamic compression was applied at either early time-points (day 1) or late time-points (day 21) of chondrogenic induction. The objective of the study is to investigate the influence of dynamic compression loading on the chondrogenic differentiation and hypertrophy of SMSCs.

## Materials and Methods

### Cell Isolation

The study was approved by the Ethical Committee of Nanjing Drum Tower Hospital, The Affiliated Hospital of Nanjing University Medical School, and informed consent was obtained from all the subjects. Synovium tissues were harvested from 13 donors (nine women and four men; mean age, 66.5 years; age range, 51–75 years) when they underwent total knee arthroplasty. The procedures for the isolation of SMSCs have been established as described previously (Shi et al., 2016). Briefly, synovium was minced, digested with type I collagenase (3 mg/mL; Gibco, United States), and passed through 70-μm nylon filter to yield single-cell suspension. The released cells were washed in PBS; resuspended in expansion medium (DMEM/F12; Gibco) containing 10% fetal bovine serum (FBS), 100 U/mL penicillin, and 100 mg/mL streptomycin (Invitrogen, United States); and plated at 5 × 10^4^ cells/cm in 100-cm^2^ culture dishes. Cells were allowed to attach for 3 days, and non-adherent cells were removed by changing the culture medium every 3 days. At day 7–10, cells were treated with trypsin–EDTA (0.25% trypsin, 1 mM EDTA; Hyclone, United States) and mixed together as passage 0 (P0). Cells were re-plated at a 1:2 dilution for subculture when cells reached confluence. Cells at passage 3 (P3) were used for all experiments.

### Fluorescence-Activated Cell Sorting Analysis

Human SMSCs at passage 3 were incubated with 1 μg of fluorescein isothiocyanate (PE)-conjugated antibodies against CD11b, CD34, CD45, CD90, and CD105 (MACS, Miltenyi Biotec, Germany). After incubation in the dark for 10 min, the cells were washed with PBS and centrifuged at 400 *g* for 10 min. Then, the stained cells were resuspended with 500 μl of ice-cold PBS and subjected to fluorescence-activated cell sorting analysis (Becton Dickinson).

### Preparation of SMSC–Agarose Constructs and Chondrogenic Induction

Cells at passage 3 were encapsulated in 2% low-melting-temperature agarose (Invitrogen, Chicago, IL, United States) at a concentration of 10 × 10^6^ cells/mL, as previously described (Mouw et al., 2007). Cell–agarose constructs were cultured in chondrogenic medium, which was high-glucose DMEM (Gibco, United States) containing 10^–7^ M dexamethasone (Sigma, United States), 50 μg/ml ascorbic acid phosphate (Sigma, United States), 100 μg/ml sodium pyruvate (Sigma, United States), 40 μg/ml proline (Sigma, United States), 1% ITS (Gibco, United States), and 10 ng/ml TGF-β3 (243-B3, R&D Systems, United States).

### Mechanical Compression System

**FIGURE 1:**
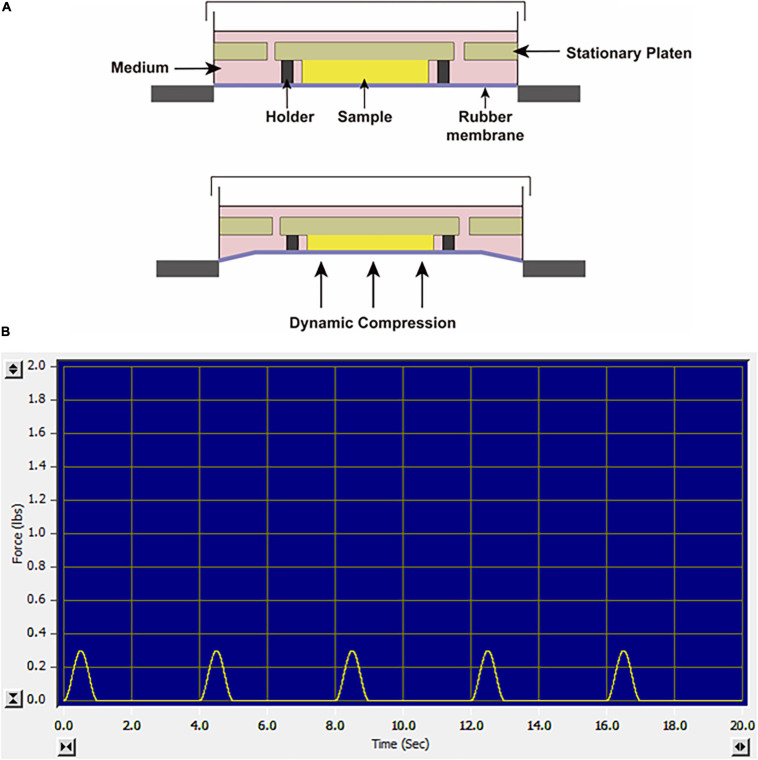
**(A)** Schematic diagram of the FX-5000^TM^ Compression System. **(B)** The screenshot of the monitor system when the constructs were exposed to sinusoidal dynamic compression at 10 kPa, 0.25 Hz.

Before loading, the SMSC–agarose constructs were placed within the 13-mm-diameter foam ring of Biopress^TM^ compression plate wells (Flexcell, United States), and 4 mL chondrogenic medium was added to each well. Dynamic compression was applied by a computer-controlled FX-5000^TM^ Compression System as described in the manufacturer’s manual. The constructs were exposed to sinusoidal dynamic compression at 10 kPa with a frequency of 0.25 Hz, and the daily regime was applied at 1 h per day. The loading scheme is shown in Figures 1A,B. The compression experiments were initiated from day 1 or day 21 in the chondrogenic differentiation period and performed for daily until day 28, which lasted for 28 days or 7 days, respectively. Cell viability was assessed by a CCK-8 kit (Dojindo, Japan) at day 28 after the final compression procedure. The CCK-8 solution was added to the culture medium of each group at the ratio of 1:10. The absorbance values were measured at 450 nm after incubation for 2 h following the manufacturer’s instruction. Unloaded groups were placed in wells but not exposed to dynamic compression.

### RNA Isolation and Real-Time Reverse Transcriptase-Polymerase Chain Reaction (q-PCR)

Cell–agarose constructs were dissolved in Qiagen lysis buffer (Qiagen, Chatsworth, CA, United States) supplemented with β-mercaptoethanol as described previously (Bougault et al., 2009). Then RNeasy Total RNA Kit was used according to the manufacturer’s protocols followed by reverse transcription using PrimeScript RT Reagent Kit (TaKaRa, Japan). Q-PCR was performed as described previously. Primer names and sequences are listed in Table 1. SYBR Green PCR Mix (Thermo Fisher Scientific, United States) was mixed with primers and cDNA, and Q-PCR was performed in ABI Step One Instruments (Applied Biosystems). The expression level of genes was calculated by the 2^–ΔΔCt^ method, and data were normalized to human β-actin gene.

**TABLE 1:** Primers names and sequences.

**Primer**	**Forward (5′-3′)**	**Reverse (3′-5′)**
Col2α1	TGGACGATCAGGCGAAACC	GCTGCGGATGCTCTCAATCT
Aggrecan	CCCCTGCTATTTCATCGACCC	GACACACGGCTCCACTTGAT
SOX9	AGCGAACGCACATCAAGAC	CTGTAGGCGATCTGTTGGGG
RUNX2	TGGTTACTGTCATGGCGGGTA	TCTCAGATCGTTGAACCTTGCTA
MMP13	ACTGAGAGGCTCCGAGAAATG	GAACCCCGCATCTTGGCTT
Col10α1	CATAAAAGGCCCACTACCCAAC	ACCTTGCTCTCCTCTTACTGC
Col1α1	AAAGATGGACTCAACGGTCTC	CATCGTGAGCCTTCTCTTGAG
ALP	TACACGGTCCTCCTATACGGAA	CTCTCGCTCTCGGTAACATC
β-actin	CATGTACGTTGCTATCCAGGC	CTCCTTAATGTCACGCACGAT

### Western Blot

Cells were recovered from the constructs at the indicated time and dissolved in lysis buffer (Thermo Fisher Scientific, United States), supplemented with inhibitors of phosphatases and proteases (Millipore, United States) as described previously (Bougault et al., 2009; Ge et al., 2019). BCA Protein Assay Kit (Pierce, United States) was used to determine the concentration of protein. Equal amount of protein was electrophoresed on a 10% Bis–Tris gel (Bio-Rad, United States) before transferring onto a nitrocellulose membrane. The membranes were then incubated with the following antibodies: SOX9 (Millipore, United States), RUNX2 (Cell signaling, United States), and GAPDH (Cell signaling, United States). All primary antibodies were applied at 1:1,000 dilution. Blots were then incubated with horseradish peroxidase (HRP)-conjugated secondary antibodies (Cell signaling, United States). The immune complexes were detected using a chemiluminescence kit (Thermo Fisher Scientific, United States) and visualized *via* the Odyssey Infrared Imaging System.

### Immunofluorescent Analysis

Immunofluorescent staining was performed to detect the accumulation of SOX9 and COL2 within the constructs. The constructs were harvested at day 28, washed with PBS, fixed in 4% paraformaldehyde at room temperature for 4 h, and rinsed with PBS. Then, the constructs were transferred to 30% sucrose overnight, frozen in OCT, and cryostat-sectioned. Sections were treated with 0.1% peroxide followed by 1% bovine serum albumin (Sigma) in PBS for blocking at room temperature. The sections were then incubated with primary antibodies at 4°C overnight. Subsequently, the sections were washed and incubated with secondary antibodies (Cell signaling, United States). The following antibodies were used: anti-SOX9 (Cell signaling, United States) and anti-COL2 (Boster, China). Then, the sections were scanned using a Leica SP5 laser confocal microscope.

### Statistical Analysis

All the data were expressed as the mean ± SD. One-way ANOVA was applied for multiple comparisons between independent groups. *P*-values less than 0.05 were considered statistically significant. SPSS 11.5 statistical software was used for statistical analysis.

## Results

### Profiles of SMSCs in Chondrogenic Induction

**FIGURE 2:**
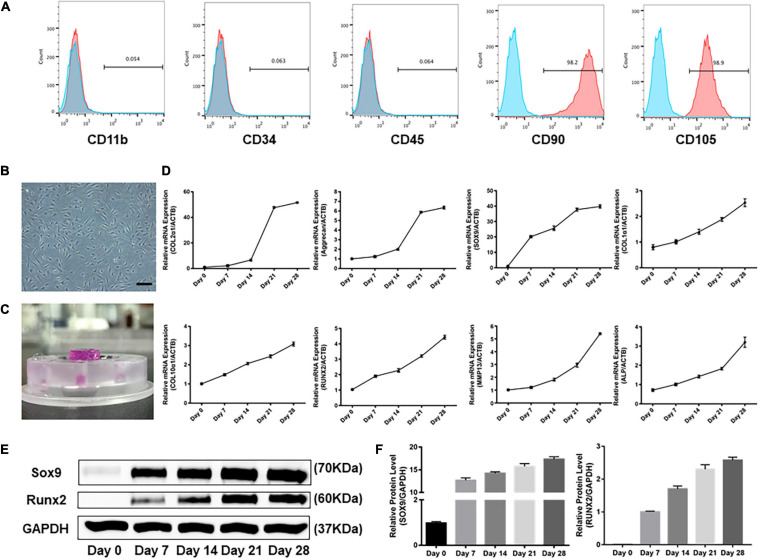
Profiles of human SMSCs in this experiment. **(A)** Flow cytometric analysis of human SMSCs at passage 3. SMSCs were positive for CD90 and CD105, while negative for CD11b, CD34, and CD45. **(B)** SMSCs at passage 3. **(C)** Patterns of SMSC constructs. **(D)** Gene expression of chondrogenesis and hypertrophy development was examined at days 0, 7, 14, 21, and 28. **(E)** Western blot analysis of expression level of SOX9 and RUNX2. **(F)** Expression level of SOX9 and RUNX2 was shown in the histograms normalized with GAPDH. COL2α1, collagen type II; SOX9, SRY-box transcription factor 9; COL1α1, collagen type I; COL10α1, collagen type X; RUNX2, RUNX family transcription factor 2; MMP13, metalloproteinases 13; ALP, alkaline phosphatase.

Flow cytometric analysis revealed that the majority of isolated cells expressed CD90 and CD105 (CD90: 98.2% and CD105: 98.9% in hSMSCs) and were negative for CD11b, CD34, and CD45 (Figure 2A). Our results showed that adherent cells exhibited a fibroblast-like morphology (Figure 2B).

As shown in Figure 2C, SMSC-agarose constructs were prepared. Transcription of ECM genes was done to analyze the profiles for chondrogenic differentiation applied in the system. The results showed that the gene expression level of collagen type II (COL2α1) and aggrecan sharply increased after being treated with TGF-β3 for 14 days, but attenuated the increase after 21 days (Figure 2D). Expression level of SRY-box transcription factor 9 (SOX9) began to spurt after 7 days but got slowly increased after 7 days (Figures 2D–F).

Hypertrophy-related genes collagen type I (COL1α1), collagen type X (COL10α1), matrix metalloproteinases 13 (MMP13), and alkaline phosphatase (ALP) exhibited a gradually increasing trend during the 28-day induction (Figure 2D). However, the mRNA and protein level of RUNX family transcription factor 2 (RUNX2) greatly increased after chondrogenic induction for 21 days (Figures 2D–F).

### Chondrogenic and Hypertrophic Gene Expression Stimulated by Dynamic Compression Without Preculture

**FIGURE 3:**
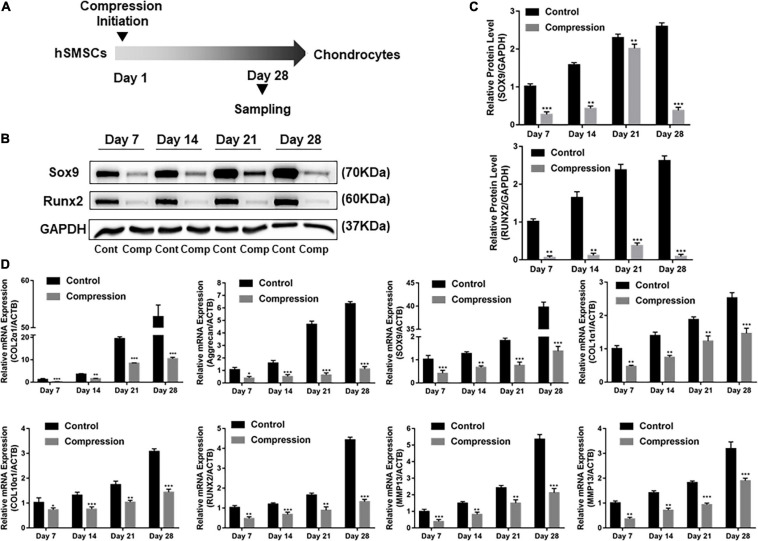
Dynamic compression without preculture inhibited both chondrogenesis and hypertrophy of SMSCs. **(A)** Schematic of the experimental protocol. **(B)** Western blot analysis of expression level of SOX9 and RUNX2 at days 7, 14, 21, and 28. **(C)** Expression level of SOX9 and RUNX2 was shown in the histograms normalized with GAPDH. **(D)** Gene expression of chondrogenesis and hypertrophy development. Results are presented as the mean ± S.D. *P < 0.05, **P < 0.01, ***p < 0.001. COL2α1, collagen type II; SOX9, SRY-box transcription factor 9; COL1α1, collagen type I; COL10α1, collagen type X; RUNX2, RUNX family transcription factor 2; MMP13, metalloproteinases 13; ALP, alkaline phosphatase.

**FIGURE 4:**
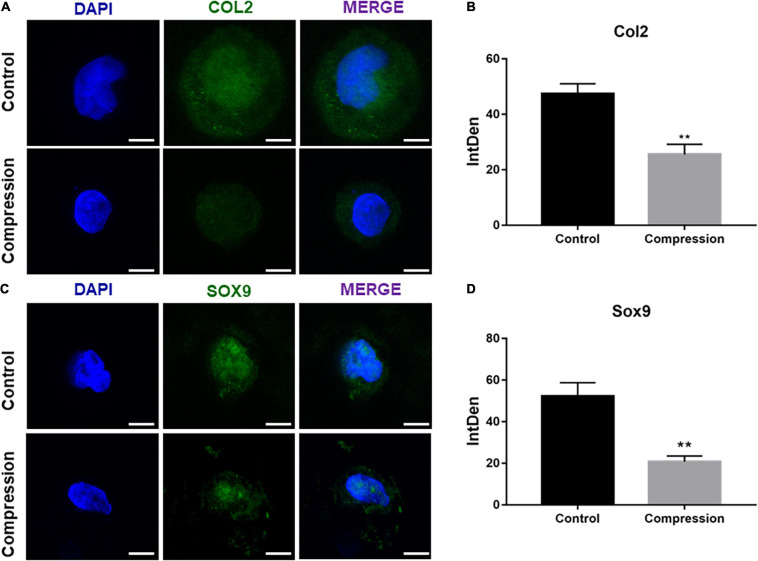
**(A,C)** Immunofluorescence staining of COL2 and SOX9 after dynamic compression without preculture at day 28. Scale bars = 10 μm. **(B,D)** Integrated density of COL2 and SOX9 by ImageJ. Results are presented as the mean ± S.D. **P < 0.01. COL2, collagen type II; SOX9, SRY-box transcription factor 9.

The SMSCs constructs were subjected to dynamic compression from the first day of chondrogenic induction (Figure 3A). The cell viability was assessed by CCK-8 after the final compression period. As shown in Supplementary Figure 1A, the viability of SMSCs was not altered after subjected to mechanical compression. Of note, significant difference was found in the expression levels of all chondrogenic genes between compression groups and unloaded controls at each time period, with the unloaded controls having the highest production of aggrecan, COL2α1, and SOX9 genes (Figures 3B–D). The chondrogenic master protein SOX9 and ECM-related collage type II (COL2) were also evaluated by immunofluorescence. The results showed that the expression of COL2 and SOX9 decreased after being exposed to mechanical compression for 28 days (Figure 4).

The effect of dynamic compression without preculture on hypertrophic development of the differentiated SMSCs was analyzed. Dynamic compression led to a significant decrease in the expression level of hypertrophic markers COL1α1, COL10α1, MMP13, and ALP (Figure 3D). For RUNX2, the expression level of groups under mechanical compression in every time-point was significantly downregulated compared with unloaded controls (Figures 3B–D).

### Chondrogenic and Hypertrophic Gene Expression Stimulated by Delayed Dynamic Compression

**FIGURE 5:**
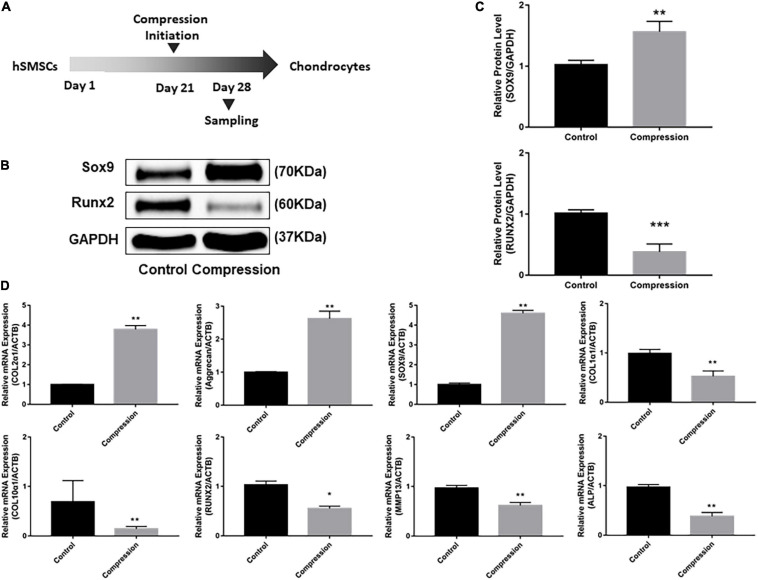
Dynamic compression applied after 3-week chondrogenic induction led to the promotion of chondrogenesis as well as the suppression of hypertrophy development of SMSCs. **(A)** Schematic of the experimental protocol. **(B)** Western blot analysis of expression levels of SOX9 and RUNX2 at day 28. **(C)** Expression levels of SOX9 and RUNX2 were shown in the histograms normalized with GAPDH. **(D)** Gene expression of chondrogenesis and hypertrophy development. Results are presented as the mean ± S.D. *P < 0.05, **P < 0.01, ***p < 0.001. COL2α1, collagen type II; SOX9, SRY-box transcription factor 9; COL1α1, collagen type I; COL10α1, collagen type X; RUNX2, RUNX family transcription factor 2; MMP13, metalloproteinases 13; ALP, alkaline phosphatase.

**FIGURE 6:**
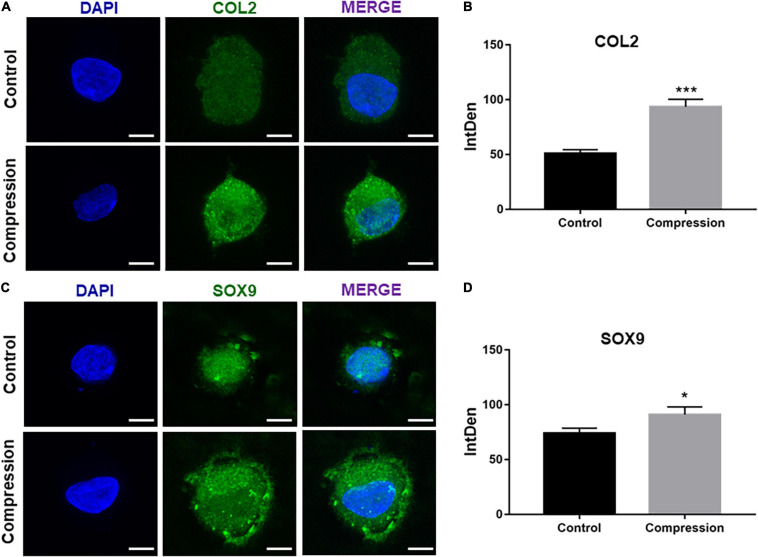
**(A,C)** Immunofluorescence staining of COL2 and SOX9 after dynamic compression applied with preculture at day 28. Scale bars = 10 μm. **(B,D)** Integrated density of COL2 and SOX9 by ImageJ. Results are presented as the mean ± S.D. *P < 0.05, ***p < 0.001. COL2, collagen type II; SOX9, SRY-box transcription factor 9.

After 3-week chondrogenic preculture, dynamic compressive stress was applied to the MSC-laden constructs and samples were harvested after 7 days of compression (Figure 5). Likewise, the application of mechanical compression did not inhibit the viability of SMSC construct (Supplementary Figure 1B). Subsequently, two trends were observed in the delayed compressive loading experiments. First, delayed dynamic compression promoted the chondrocyte marker gene expression. Expression level of SOX9 was found to increase following mechanical compression (Figures 5B,C). Correspondingly, the mRNA level of aggrecan in loaded specimens was higher compared with their unloaded counterparts. Similarly, delayed mechanical compression resulted in higher COL2α1 expression in mRNA levels (Figure 5D). COL2 immunostaining demonstrated that dynamic compression for 7 succession days led to an increase in the accumulation of COL2 matrix around the SMSCs (Figure 6). Second, delayed dynamic compression was found to significantly suppress the expression of hypertrophic marker during chondrogenic differentiation. The mRNA levels of COL1α1, COL10α1, MMP13, RUNX2, and ALP in unloaded groups increased steadily from day 1 to day 28 (Figures 2D–F). Comparatively, under delayed dynamic compression conditions, the expression of COL1α1, COL10α1, MMP13, and ALP was significantly suppressed compared with unloaded counterparts in day 28 (Figure 5D). Similarly, the expression level of RUNX2 significantly downregulated in response to delayed dynamic compression (Figures 5B–D).

## Discussion

Numerous diseases may lead to cartilage damage or OA, which is a worldwide clinical problem (Muir, 1995). Although efforts have been made for cartilage repair and approaches such as ACI and microfracture show great prospects in clinical therapy, these treatments stills do not lead to the full regeneration of cartilage in its native form (Bedi et al., 2010). MSCs have become an ideal selection for the regeneration of cartilage, and several studies have been performed with different kinds of MSCs such as BMSCs. Although BMSCs show great potentials in bone regeneration, the ability of BMSCs for cartilage regeneration remains limited considering the fact that BMSCs display a much great tendency for osteogenesis during chondrogenic differentiation (Djouad et al., 2005; Pelttari et al., 2006; De Bari et al., 2008). SMSCs is a promising cell source for cartilage tissue engineering given their strong potential to undergo chondrogenic differentiation (Pei et al., 2008). The superiority of SMSCs for cartilage repair is as follows. First, the gene expression profile of chondrocytes resembles SMSCs as it showed that they all express COL2α1, and both chondrocytes and SMSCs share the same pool of precursor cells. The gene profile of SMSCs matches the chondrocytes more closely than that of BMSCs (Horie et al., 2009). Second, SMSCs possess a more prominent colony-forming potential than BMSCs (Jo et al., 2007). Third, the acquisition of SMSCs in clinical fields is appealing in that synovium in the joint can be obtained with fewer complications and minimal invasiveness compared with the isolation of other types of MSCs such as BMSCs or adipose-derived stem cells (ASCs) (Sakaguchi et al., 2005). Considering all these advantages and great potentials of SMSCs in cartilage repair, we applied the SMSCs for further studies.

It was reported that chondrogenic differentiation of MSCs toward hypertrophy that it was inevitable *in vitro* (Pelttari et al., 2006; Yang et al., 2012). Several lines of evidence showed that SMSCs displayed a much lighter potential for chondrogenic hypertrophy in pellet culture (Pei et al., 2008). However, in accordance with previous reports, our results showed that SMSC constructs in unloaded conditions were not maintained *in vitro* culture for 4 weeks, even in the presence of TGF-β, which indicated that further optimizations may be required to generate SMSC-seeded constructs akin to that of articular neo-cartilage. Cartilage in our body is exposed to a large range of mechanical stresses (Holmvall et al., 1995; Gemmiti and Guldberg, 2006; Verteramo and Seedhom, 2007). Mechanical stimulation has been reported to show great application prospect in regulating MSC chondrogenic differentiation (Huang et al., 2010; Zhang et al., 2015). The present study was aimed to determine the feasibility of mechanical compression to promote chondrogenic differentiation of SMSCs and maintain the chondrocyte phenotype.

First, we applied dynamic mechanical compression daily to SMSC constructs from the first day of chondrogenic induction. The results showed that dynamic compression without preculture inhibited chondrogenic differentiation of SMSCs. This is in line with numerous studies showing the detrimental effect of mechanical compression on chondrogenic differentiation of MSCs without preculture (Thorpe et al., 2008, 2010; Huang et al., 2010). Huang et al. (2010) reported that continuous loading which initiated immediately following the construct creation resulted in a negative effect of sGAG and collagen content. Another study showed that loading without preculture led to a decrease in sGAG content (Thorpe et al., 2010). A number of factors may explain the results observed. Although mechanical compression promoted cartilage-related gene expression and protein synthesis in chondrocyte constructs, SMSCs may not respond to mechanical stimulation at the early stage of the differentiation process in a similar manner. Mouw et al. (2007) observed that mechanical stress applied to MSCs after 16 days increased COL2α1 and aggrecan gene expression or protein synthesis, while loading in the early time-points did not alter all these measurements. Huang et al. (2010) reported that preculture for a relatively long time (21 days) led to a significant increase in COL2α1 and aggrecan expression compared with those constructs that precultured for a relatively short time. Likewise, chondrocytes in agarose gels under mechanical stress may synthesize more cartilage matrix at later time-points than early, indicating that a mature and developed matrix was required for transduction from mechanical compression to biochemical signals (Buschmann et al., 1995). Also, the mechanical compression was applied from day 7 or day 14 daily until day 28, the final day of chondrogenic differentiation during our observation period. Likewise, as displayed in Supplementary Figures 2, 3, our results showed that mechanical compression from either day 7 or day 14 exerted an inhibitory effect of chondrogenesis. However, despite the unwanted inhibition in chondrogenesis, mechanical compression dramatically resulted in a significant decrease in the expression of hypertrophic genes such as COL1α1, COL10α1, RUNX2, MMP13, and ALP, implying that mechanical stress may inhibit hypertrophy of chondrocytes during chondrogenic induction.

To find out the appropriate initial time for dynamic compression, we examined the profile of SMSCs under chondrogenic induction by analyzing chondrocyte-specific markers. Our results revealed that ECM gene transcription increased quickly after 2-week chondrogenic differentiation but slowed down after 3 weeks. Although the expression level of ECM genes seemed to reach a plateau, a relative slight but constant increase was also observed from day 21 to day 28. Meanwhile, hypertrophy-related gene transcription increased gradually during 3-week chondrogenic induction but began to spurt after 3 weeks. Therefore, in the following study, we delayed the application of dynamic compression for 3 weeks, and the application of compression was initiated from day 21, hoping for a further promotion in chondrogenesis as well as a suppression in hypertrophy.

Another major concern of long-term MSC culture is the hypertrophic differentiation and ossification during chondrogenic induction (Pelttari et al., 2006; Bian et al., 2012). Our results showed that delayed dynamic compression promoted the gene expression of aggrecan and COL2α1 as well as the expression level of chondrogenic master gene SOX9, which is in line with various studies showing the favorable effect of delayed dynamic compression on the differentiation of MSCs, when the parameters were up to 4 h per day within 0.1–1 Hz of frequency of dynamic stimulation (Huang et al., 2010; Li et al., 2012; Zhang et al., 2015). In our study, we showed that under our stimulation parameters, the increase in ECM gene expression with delayed dynamic compression was accompanied by a suppression of hypertrophy during chondrogenic differentiation. Contrasting results on hypertrophic differentiation under the stimulation of mechanical compression have been reported. Huang et al. (2010) reported that long-term dynamic compressive stress led to an increase in the expression of hypertrophic genes. Li et al. (2012) reported that higher expression of COL10α1 was noticed under the stimulation of axial compressive stress in the low dose of TGF-β. In these studies, dynamic compression was applied in the absence or low dose of TGF-β. On the other hand, other studies reported that dynamic compression contributed to the suppression of hypertrophy after chondrogenic induction in the presence of high dose of TGF-β, which is similar to our study (Zhang et al., 2015). These outcomes above indicated that the reaction of MSCs to dynamic compression depended on the parameters of the loading regime, including dose of growth factors, frequency, duration, and intensity, as well as types of scaffolds and differentiation stage of MSCs.

In our experiments, the exogenous cytokine TGF-β3 was added in the induction medium during the full period of experiment. Although previous studies reported that dynamic compression may exert a pro-chondrogenic effect and increase the accumulation of sGAG, TGF-β3 alone led to far greater accumulation of sGAG as well as collagen content compared with stimulation with mechanical compression alone (Kisiday et al., 2009). Moreover, the chondrogenic effect induced by mechanical compression in the absence of exogenous cytokine appeared to be transient and the expression level of COL2α1 and aggrecan returned to the baseline shortly after the removal of the exogenous cytokine TGF-β3 (Huang et al., 2010). Of note, we also tried to replace TGF-β3 by mechanical compression in our preliminary experiments considering the cost of this kind of expensive cytokine. The SMSC constructs were allowed to undergo chondrogenic differentiation until day 21. Subsequently, TGF-β3 was withdrawn in half numbers of the constructs, and the constructs subjected to compression or control were further divided into TGF-β3-continued group and TGF-β3-discontinued group. Dramatically, in accordance with the previous report, the expression of chondrogenic marker as COL2α1, aggrecan, and SOX9 sharply decreased after the removal of exogenous cytokine TGF-β3. Although mechanical compression alone did induce an increase in the expression level of pro-chondrogenic genes, the TGF-β3 led to a far greater increase in pro-chondrogenic genes. Moreover, in the presence of mechanical compression, TGF-β3 led to an increase in the expression level of pro-chondrogenic genes. Besides, considering the hypertrophic markers, mechanical compression exerted an anti-hypertrophy effect regardless of the presence or absence of TGF-β3 (Supplementary Figure 3).

Our study has several limitations. First, it was reported that several signaling pathways have been involved in the regulation of chondrogenesis under the stimulation of mechanical compression, including mitogen-activated protein kinase (MAPK), insulin-like factor-1 (IGF-1), and TGF-β signaling pathways (Li et al., 2012). Among them, TGF-β/SMAD plays a dominant role in the development of cartilage and maintenance of cartilage phenotype (Wang et al., 2014). The downstream target of TGF-β/SMAD includes SMAD2/3 and SMAD1/5/8. SMAD2/3 mainly participates in the chondrogenesis, while SMAD1/5/8 is involved in the chondrogenic hypertrophy. Previous report had demonstrated that mechanical compression promoted the phosphorylation of SMAD2/3, while it suppressed the phosphorylation of SMAD1/5/8 (Hellingman et al., 2011). Our results showed that mechanical compression promoted the chondrogenesis in a time-dependent manner and compression initiated before preculture with TGF-β3 for 3 weeks led to the inhibitory effect of chondrogenic differentiation, which may imply that the effect of mechanical stress on phosphorylation of SMAD2/3 is also dependent on the period of chondrogenesis. In future studies, a robust research about the regulation of mechanical compression on the phosphorylation of SMAD2/3 would be conducted. Second, the SMSCs in our study were isolated from the synovium tissues of patients with OA who underwent total knee arthroplasty. It was reported that the ability to differentiate into chondrocytes and proliferate varied from different donors, and MSCs from patients with OA may be surrounded by different kinds of inflammatory factors such as interleukin-1β or tumor necrosis factor-α, which may interfere with the chondrogenesis of SMSCs (García-Álvarez et al., 2011). Synovium obtained from patients who undergo meniscectomy or anterior cruciate ligament (ACL) reconstruction arthroscopically may be better source for the isolation of SMSCs.

Taken together, our results suggest that the applications of mechanical compression have a significant influence on both the chondrogenic and hypertrophic differentiation of SMSCs, and that allowing for a period of chondrogenic induction produced a tissue which reacted more positively to mechanical compression. To our knowledge, this is the first study to demonstrate the beneficial effect of dynamic compression to promote the stabilization of chondrocyte phenotype in SMSCs. Our findings also give new insight into the important implications of mechanical compression to postoperative rehabilitation management following clinical cell-based treatment for cartilage replacement and regeneration. Future studies are required to further elucidate the specific underlying mechanism involving the role mechanical stimulation plays in the regulation of chondrogenic differentiation of MSCs.

## Data Availability Statement

The raw data supporting the conclusions of this article will be made available by the authors upon reasonable request, without undue reservation.

## Ethics Statement

The studies involving human participants were reviewed and approved by the Ethics Committee of Nanjing Drum Tower Hospital, The Affiliated Hospital of Nanjing University Medical School. The patients/participants provided their written informed consent to participate in this study.

## Author Contributions

YG and YL wrote the manuscript. YL and ZW collected and analyzed the data. LL revised the manuscript. LL, HT, and QJ designed and supervised the study. All authors read and approved the final manuscript.

## Conflict of Interest

The authors declare that the research was conducted in the absence of any commercial or financial relationships that could be construed as a potential conflict of interest.

## Supplementary Material

Supplementary Figure 1 **(A)** Relative cell viability of SMSC construct after subjected to dynamic compression from day 1. **(B)** Relative cell viability of SMSC construct after subjected to dynamic compression applied after 3-week chondrogenic induction.

Supplementary Figure 2 Dynamic compression applied after 1-week chondrogenic induction inhibited both chondrogenesis and hypertrophy development of SMSCs. Gene expression of chondrogenesis and hypertrophy development. Results are presented as the mean ± S.D. **P* < 0.05, ***P* < 0.01, ****p* < 0.001. COL2α1, collagen type II; SOX9, SRY-box transcription factor 9; COL1α1, collagen type I; COL10α1, collagen type X; RUNX2, RUNX family transcription factor 2; MMP13, metalloproteinases 13; ALP, alkaline phosphatase.

Supplementary Figure 3 Dynamic compression applied after 2-week chondrogenic induction inhibited both chondrogenesis and hypertrophy development of SMSCs. Gene expression of chondrogenesis and hypertrophy development. Results are presented as the mean ± S.D. **P* < 0.05, ***P* < 0.01, ****p* < 0.001. COL2α1, collagen type II; SOX9, SRY-box transcription factor 9; COL1α1, collagen type I; COL10α1, collagen type X; RUNX2, RUNX family transcription factor 2; MMP13, metalloproteinases 13; ALP, alkaline phosphatase.

Supplementary Figure 4 Gene expression of chondrogenesis and hypertrophy development after retraction of TGF-β3. The SMSC constructs were allowed to undergo chondrogenic differentiation until day 21. Subsequently, TGF-β3 was withdrawn in half of the constructs, and the constructs subjected to compression or control were further divided into TGF-β3-continued group and TGF-β3-discontinued group. Results are presented as the mean ± S.D. **P* < 0.05, ***P* < 0.01, ****p* < 0.001. COL2α1, collagen type II; SOX9, SRY-box transcription factor 9; COL10α1, collagen type X; RUNX2, RUNX family transcription factor 2; MMP13, metalloproteinases 13.
