# Supplementary figures and images for: Effects of Mechanical Compression on Chondrogenesis of Human Synovium-Derived Mesenchymal Stem Cells in Agarose Hydrogel
Source: Front Bioeng Biotechnol. 2021 Jul 19;9:697281. doi: 10.3389/fbioe.2021.697281 (PMC8327094; doi:10.3389/fbioe.2021.697281)

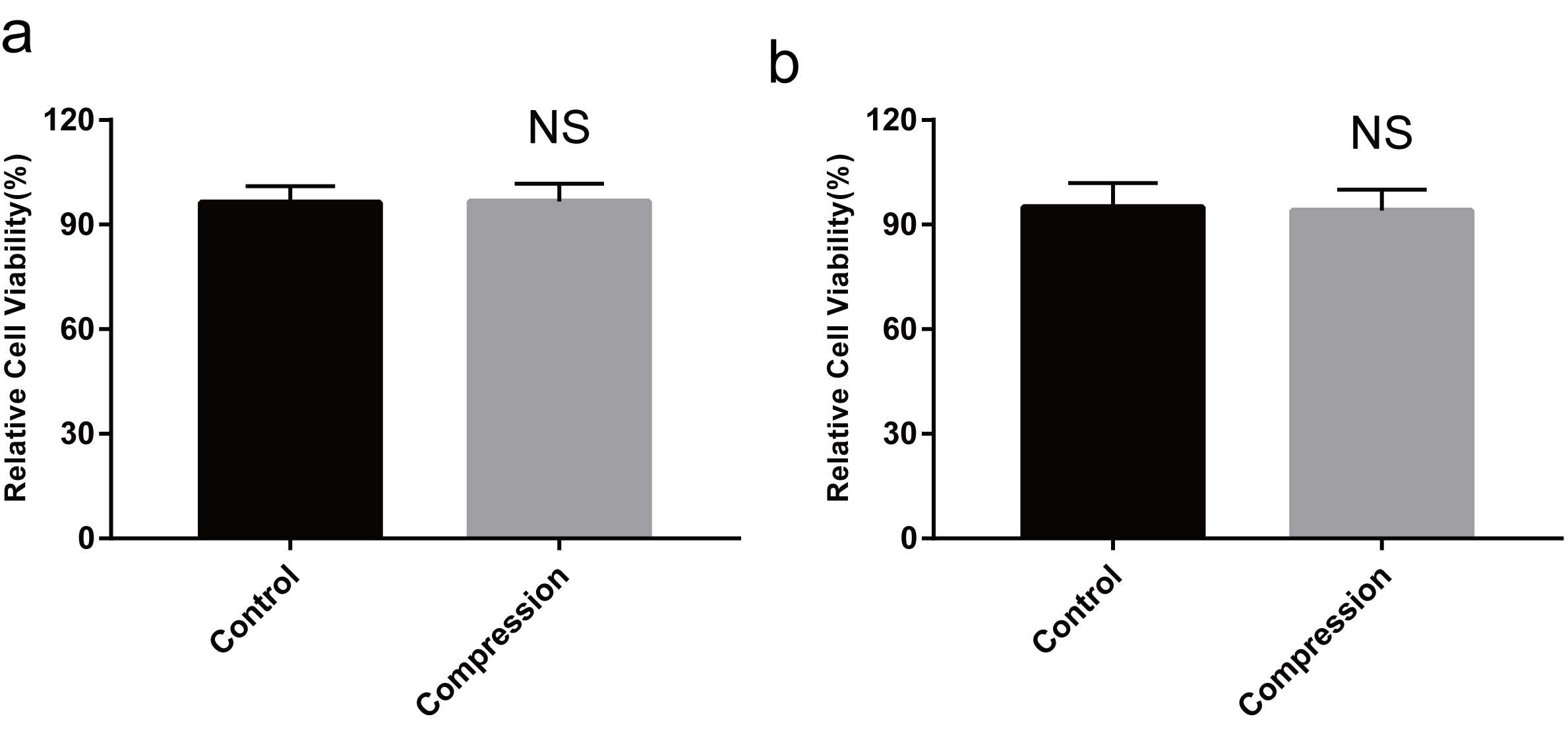

Supplement: Supplementary Figure 1 — (A) Relative cell viability of SMSC construct after subjected to dynamic compression from day 1. (B) Relative cell viability of SMSC construct after subjected to dynamic compression applied after 3-week chondrogenic induction. [file Image_1.TIF]

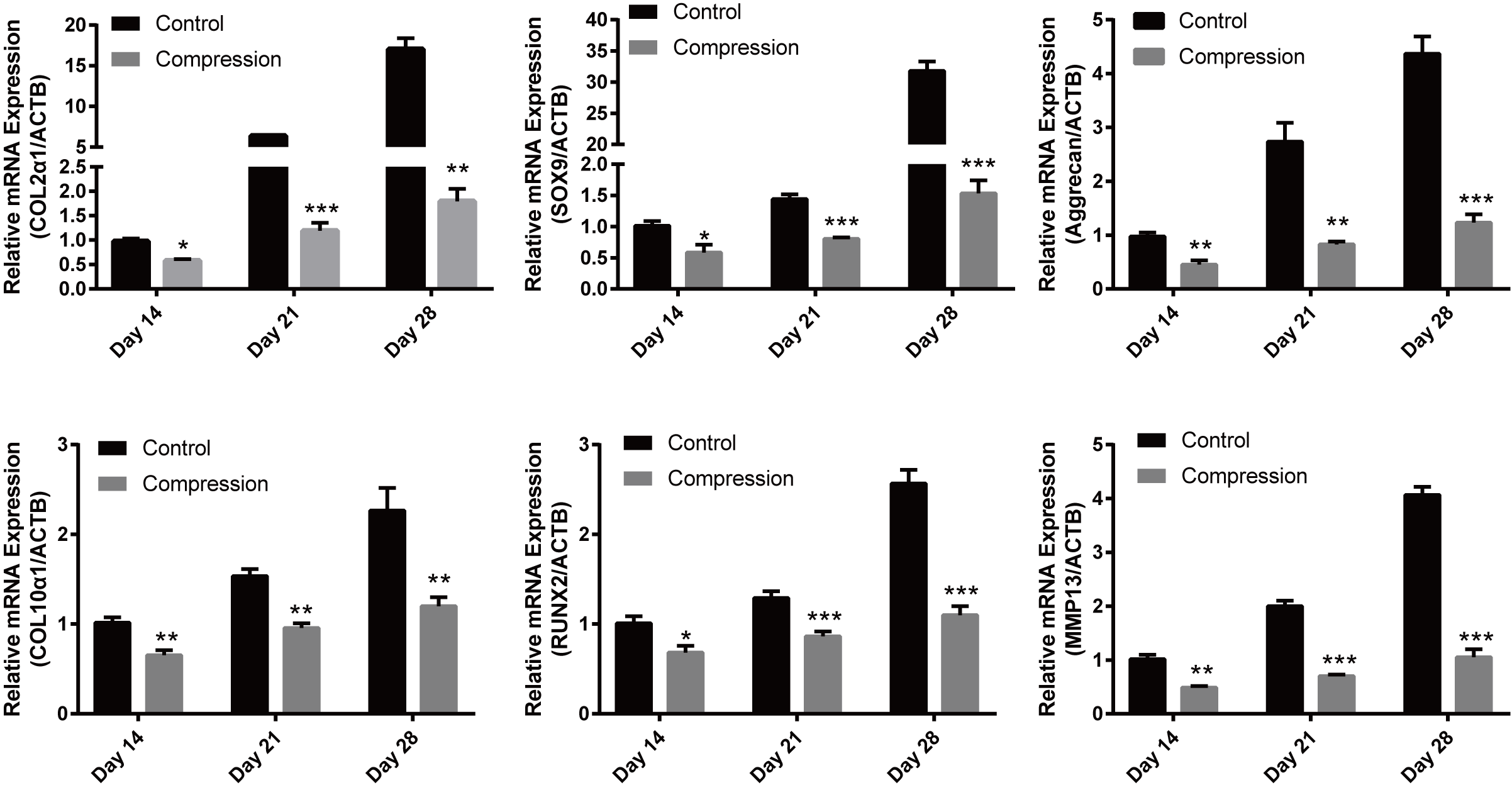

Supplement: Supplementary Figure 2 — Dynamic compression applied after 1-week chondrogenic induction inhibited both chondrogenesis and hypertrophy development of SMSCs. Gene expression of chondrogenesis and hypertrophy development. Results are presented as the mean ± S.D. *P < 0.05, **P < 0.01, ***p < 0.001. COL2α1, collagen type II; SOX9, SRY-box transcription factor 9; COL1α1, collagen type I; COL10α1, collagen type X; RUNX2, RUNX family transcription factor 2; MMP13, metalloproteinases 13; ALP, alkaline phosphatase. [file Image_2.TIF]

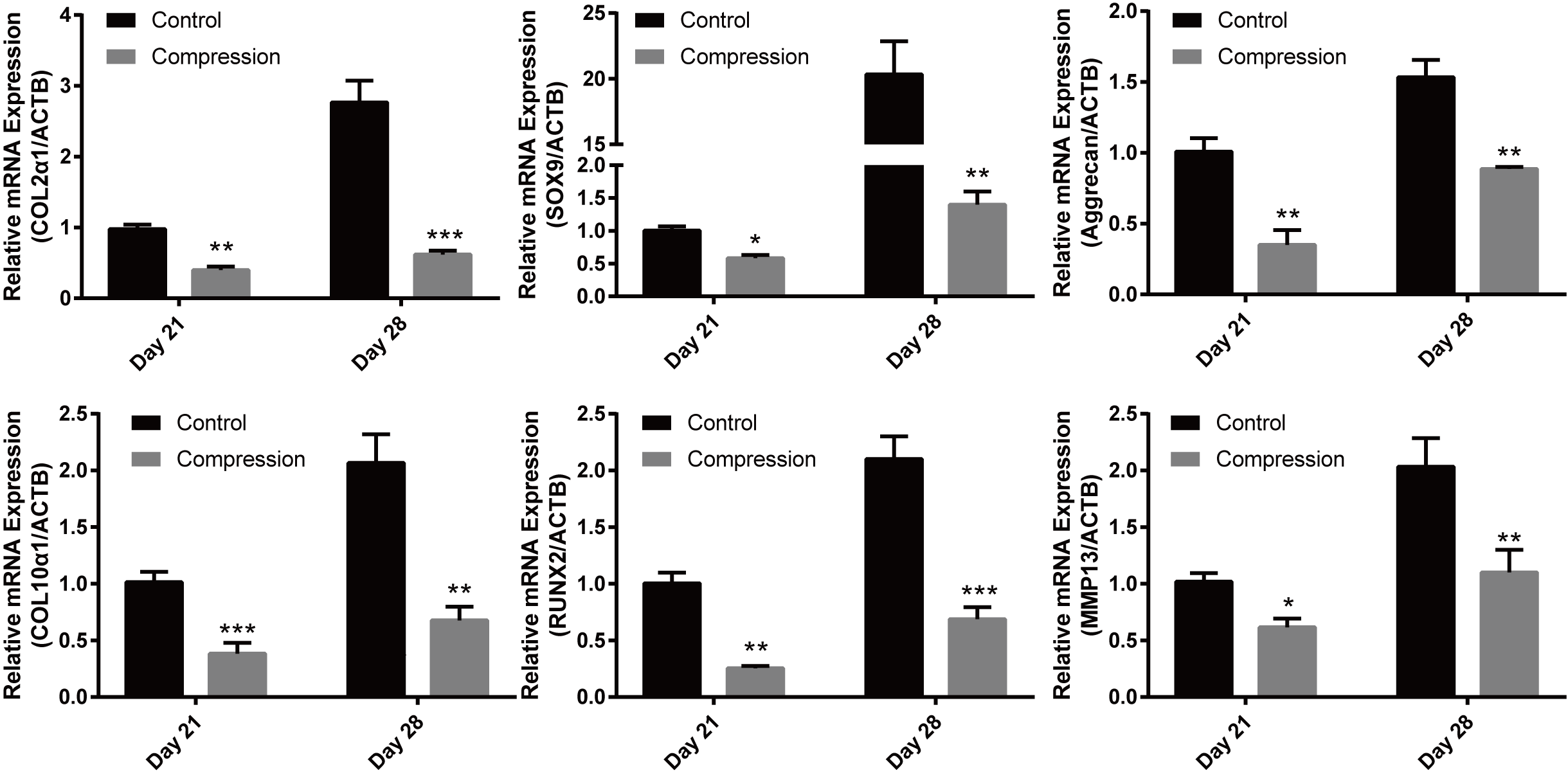

Supplement: Supplementary Figure 3 — Dynamic compression applied after 2-week chondrogenic induction inhibited both chondrogenesis and hypertrophy development of SMSCs. Gene expression of chondrogenesis and hypertrophy development. Results are presented as the mean ± S.D. *P < 0.05, **P < 0.01, ***p < 0.001. COL2α1, collagen type II; SOX9, SRY-box transcription factor 9; COL1α1, collagen type I; COL10α1, collagen type X; RUNX2, RUNX family transcription factor 2; MMP13, metalloproteinases 13; ALP, alkaline phosphatase. [file Image_3.TIF]

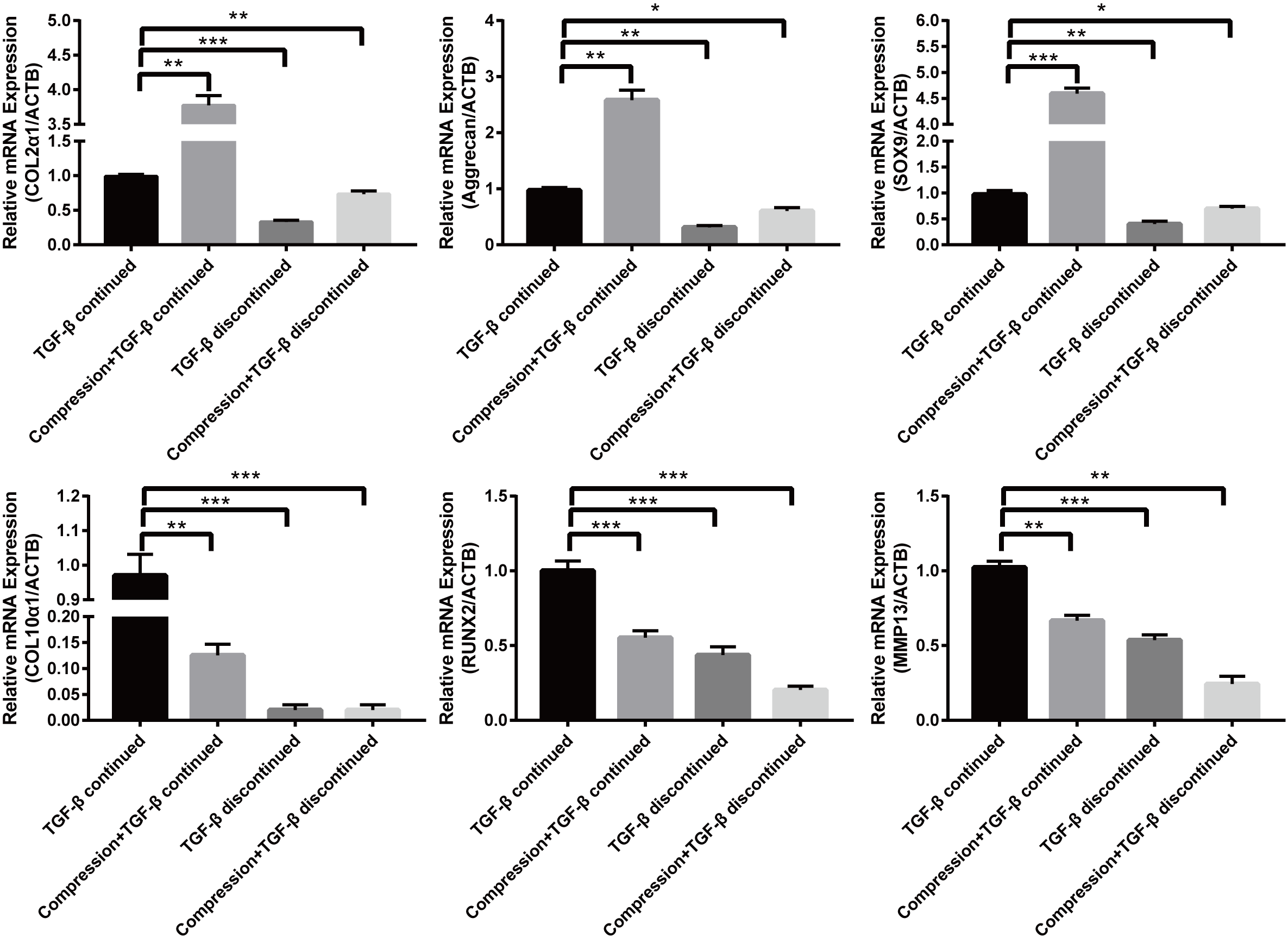

Supplement: Supplementary Figure 4 — Gene expression of chondrogenesis and hypertrophy development after retraction of TGF-β3. The SMSC constructs were allowed to undergo chondrogenic differentiation until day 21. Subsequently, TGF-β3 was withdrawn in half of the constructs, and the constructs subjected to compression or control were further divided into TGF-β3-continued group and TGF-β3-discontinued group. Results are presented as the mean ± S.D. *P < 0.05, **P < 0.01, ***p < 0.001. COL2α1, collagen type II; SOX9, SRY-box transcription factor 9; COL10α1, collagen type X; RUNX2, RUNX family transcription factor 2; MMP13, metalloproteinases 13. [file Image_4.TIF]
